# *Bacillus pumilus* Septic Arthritis in a Healthy Child

**DOI:** 10.1155/2016/3265037

**Published:** 2016-04-24

**Authors:** V. M. Shivamurthy, Soren Gantt, Christopher Reilly, Peter Tilley, Jaime Guzman, Lori Tucker

**Affiliations:** ^1^Division of Paediatric Rheumatology, BC Children's Hospital, University of British Columbia, Vancouver, BC, Canada V6H 3V4; ^2^Division of Infectious Diseases, BC Children's Hospital, University of British Columbia, Vancouver, BC, Canada V6H 3V4; ^3^Department of Paediatric Orthopaedics, BC Children's Hospital, University of British Columbia, Vancouver, BC, Canada V6H 3V4; ^4^Department of Pathology and Laboratory Medicine, BC Children's Hospital, University of British Columbia, Vancouver, BC, Canada V6H 3V4

## Abstract

We report a case of septic arthritis caused by a* Bacillus* species,* B. pumilus*, occurring in a healthy child. This organism rarely causes serious infections and has only been described in newborns and immunocompromised individuals or as a skin infection. This child developed an indolent joint swelling after a minor skin injury, and symptoms were initially thought most consistent with chronic arthritis. The case demonstrates that clinicians should consider joint infection in children presenting with acute monoarticular swelling, even without prominent systemic features.

## 1. Introduction

Septic arthritis occurs in children at an incidence of 5–12/100,000 [1, 2]. The most common pathogenic organisms are* Staphylococcus aureus*,* Streptococcus pyogenes*, and* Kingella kingae*, which infect the joint primary through haematogenous spread [3].* Bacillus* species can cause a range of infections, including food poisoning (*B. cereus*), anthrax (*B. anthracis*), and hardware infections. However,* Bacillus* species are a frequent laboratory contaminant and appear rarely to cause septic arthritis. Here we describe, to our knowledge, the first report of septic arthritis in a healthy child due to* Bacillus pumilus*.

## 2. Case Report

A 6-year-old previously healthy Caucasian girl presented with history of pain and swelling in her left knee for 5 weeks. Her symptoms began while on holiday with her family in South Africa, when she fell while running. She developed pain and swelling of the left knee and was unable to weight bear. Her parents took her to the local emergency room, where she was found to have a swollen left knee, a small superficial abrasion over the knee, but no other external injuries. There was no radiological evidence of fracture. In the first few days following the fall, she had a low-grade fever and brief decrease in appetite. Management was conservative, with analgesics and a splint for 2 weeks. Her systemic symptoms resolved but the knee swelling and pain persisted.

On returning to Canada, three weeks after the fall, she was taken to our hospital's emergency room where repeat X-ray showed prepatellar soft tissue swelling and superior pole changes at the patella that could represent either a healing fracture or a normal variant. Rest and nonsteroidal antiinflammatory treatment were recommended.

There was slow improvement in the next few days but upon resuming physical activities the left knee pain and swelling worsened. She was seen in the orthopedic clinic at the BC Children's Hospital, where a large left knee effusion was found with no overlying redness; there was limited range of motion and mild quadriceps wasting. The complete blood count showed haemoglobin of 109 g/L, white blood count of 6.9 × 10^9^/L, platelets of 528 × 10^9^/L, ESR of 66 mm/hr, and CRP of 28 mg/L. Repeat X-ray showed marked soft tissue swelling anterior to the knee as well as within the knee joint and a possible healing fracture at the superior pole of the patella. Subsequent MRI of her left knee showed a large effusion, a radial tear of the medial meniscus, and a bipartite patella that was felt to be a normal variant with no evidence of fracture.

She was subsequently referred to the paediatric rheumatology team by the paediatric orthopaedic consultant, five weeks after her initial fall, for evaluation of suspected chronic arthritis. At that time, she had persistent left knee swelling and limp as well as pain with weight bearing. She was otherwise well and had remained afebrile. On examination, she was a cheerful and well-appearing child with a normal general physical examination. On musculoskeletal examination the sole abnormality was involving the left knee, which was warm and had a large effusion. There was a flexion deformity of 20 degrees and attempts at further extension were very painful. A differential diagnosis of early juvenile idiopathic arthritis versus chronic atypical septic arthritis was considered.

A knee aspiration and synovial biopsy were done under radiological guidance. The synovial fluid showed 27,450 × 10^6^/L white cells which were predominantly neutrophils. The biopsy showed evidence of acute and chronic inflammation with few Gram-positive bacteria on Gram stain. The acid-fast bacilli stain was negative. Cultures from the joint grew only* Bacillus pumilus*, as determined by mass spectrometry (BioTyper 3.1 software with the database update 5627, Bruker MicroFlex LT, Milton, ON) with a score of 2.281; no other species had high scores, including the closely related* B. safensis*. The* B. pumilus* was considered likely to be a contaminant. The patient was commenced on oral cephalexin for empirical coverage of common possible causes of septic arthritis after consulting with the Infectious Diseases team.

However, joint swelling and pain worsened and laboratory testing showed a WCC of 9.1 × 10^9^/L, an ESR of 35 mm/hr, and a CRP of 12 mg/L. A week after the initial aspiration, she underwent an arthroscopy which resulted in the release of a large amount of pus under pressure. A culture again grew* B. pumilus* identified by mass spectrometry, and a 16S rDNA sequence confirmed a* Bacillus* species, not* cereus* or* anthracis*. Susceptibility testing showed sensitivity to ciprofloxacin, vancomycin, septra, and imipenem.

Infectious Diseases reconsultation was requested, and the team recommended treatment with intravenous vancomycin. The knee swelling and pain improved steadily while on antibiotic therapy with a rapid decrease of the CRP. The patient developed neutropenia after 2 weeks of treatment that was attributed to vancomycin, and therefore she was switched to oral ciprofloxacin for an additional 4-week course. Her neutropenia resolved and she continued to improve. At the time of last assessment, 5 months after the initial fall, she was fully functional and parents denied any restriction of activities. Her left knee had almost full range of motion with no tenderness or effusion. The ESR and CRP had normalized.

## 3. Discussion

*Bacillus pumilus* is a Gram-positive, aerobic, spore-forming* Bacillus*, usually found in the soil as a commensal and more commonly isolated in cultures as contaminants, but rarely implicated as a pathogen.* B. pumilus* has been reported to have caused sepsis in the newborns and the immunocompromised [4, 5], central venous catheter infections [6], and cutaneous infections [7]. Septic arthritis due to* Bacillus* species has been reported after surgery in an elderly woman [8], and as far as we are aware, septic arthritis from* Bacillus* species has not previously been reported in healthy young adults or children.

In our case, the course was indolent and fever was never a prominent symptom. Pain and swelling in the knee were the main symptoms, with elevated inflammatory markers in blood. The patient's clinical presentation would have been otherwise consistent with juvenile idiopathic arthritis, but given the subacute presentation, we felt that infection needed to be ruled out. The joint aspiration was a critical procedure which provided the diagnosis in this case.* B. pumilus* was likely to be the cause of arthritis given that bacterial growth in synovial fluid was confirmed on more than one occasion. Of note, mass spectrometry can distinguish* Bacillus* species with high confidence, while 16S rDNA genotyping is not sufficient and sequencing requires inclusion of the gyrB and rpoB regions [9]. The diagnosis is further supported by the complete resolution of symptoms after treatment with appropriate antibiotics. However, we cannot exclude the possibility that other pathogens not identified contributed to a polymicrobial infection. We suspect the initial trauma may have introduced bacteria into the joint, perhaps as a result of a small penetrating injury that was not clinically apparent, akin to plant thorn synovitis [10]. Although there is most experience with treatment of* B. pumilus* infections with intravenous vancomycin, quinolones also appear to be effective [11, 12]. Vancomycin-induced neutropenia is uncommon and has usually been associated with treatment longer than 2 weeks [13, 14]. In our reported case, although the neutrophil count quickly recovered once vancomycin was discontinued, we cannot be certain of the causality. A prolonged course of therapy was given due to the chronic nature and atypical infectious cause of her arthritis, as well as the published experience with* Bacillus* bone and joint infections [15–17].

## 4. Conclusions

Septic arthritis due to atypical organisms should be considered high in the differential diagnosis when frank arthritis develops in a single joint subacutely with high inflammatory markers, even in the absence of prominent fever. Growth of* Bacillus pumilus* or other possible contaminants from normally sterile sites should not be automatically disregarded as a contaminant, especially when the patient's presentation and course are atypical.
